# Characterization of *Ligilactobacillus salivarius* CRISPR-Cas systems

**DOI:** 10.1128/msphere.00171-24

**Published:** 2024-07-11

**Authors:** Avery Roberts, Daniel Spang, Rosemary Sanozky-Dawes, Matthew A. Nethery, Rodolphe Barrangou

**Affiliations:** 1 Department of Food, Bioprocessing and Nutrition Sciences, North Carolina State University, Raleigh, North Carolina, USA; The University of Iowa, Iowa City, Iowa, USA

**Keywords:** *Ligilactobacillus*, salivarius, CRISPR, Cas

## Abstract

*Ligilactobacillus* is a diverse genus among lactobacilli with phenotypes that reflect adaptation to various hosts. CRISPR-Cas systems are highly prevalent within lactobacilli, and *Ligilactobacillus salivarius*, the most abundant species of *Ligilactobacillus*, possesses both DNA- and RNA-targeting CRISPR-Cas systems. In this study, we explore the presence and functional properties of I-B, I-C, I-E, II-A, and III-A CRISPR-Cas systems in over 500 *Ligilactobacillus* genomes, emphasizing systems found in *L. salivarius*. We examined the I-E, II-A, and III-A CRISPR-Cas systems of two *L. salivarius* strains and observed occurrences of split *cas* genes and differences in CRISPR RNA maturation in native hosts. This prompted testing of the single Cas9 and multiprotein Cascade and Csm CRISPR-Cas effector complexes in a cell-free context to demonstrate the functionality of these systems. We also predicted self-targeting spacers within *L. salivarius* CRISPR-Cas systems and found that nearly a third of *L. salivarius* genomes possess unique self-targeting spacers that generally target elements other than prophages. With these two *L. salivarius* strains, we performed prophage induction coupled with RNA sequencing and discovered that the prophages residing within these strains are inducible and likely active elements, despite targeting by CRISPR-Cas systems. These findings deepen our comprehension of CRISPR-Cas systems in *L. salivarius*, further elucidating their relationship with associated prophages and providing a functional basis for the repurposing of these Cas effectors for bacterial manipulation.

**IMPORTANCE:**

*Ligilactobacillus salivarius* is a diverse bacterial species widely used in the food and dietary supplement industries. In this study, we investigate the occurrence and diversity of their adaptive immune systems, CRISPR-Cas, in over 500 genomes. We establish their function and provide insights into their role in the interplay between the bacterial host and the predatory phages that infect them. Such findings expand our knowledge about these important CRISPR-Cas immune systems widespread across the bacterial tree of life and also provide a technical basis for the repurposing of these molecular machines for the development of molecular biology tools and the manipulation and engineering of bacteria and other life forms.

## INTRODUCTION

As a genus recently segregated from the broad *Lactobacillus* group, *Ligilactobacillus* is a group of lactic acid bacteria comprised of members with ecologically diverse niches (1). *Ligilactobacillus* strains, mainly consisting of *Ligilactobacillus salivarius*, have been isolated from various vertebrate hosts and serve as commensal members of the human gut and oral microbiota (2). The survivability of these strains in the gastrointestinal tract of their hosts has led to their broad use as an industrially relevant probiotic (3). *Ligilactobacillus* genomes are unusual among lactobacilli and often contain genomes with multiple plasmids, including megaplasmids spanning over 200 kb in size (4). These plasmids often encode for a range of genes enhancing the metabolic potential of the host strain, such as bile salt hydrolases and bacteriocins (4). As identified before taxonomic reclassification, some *Ligilactobacillus* strains also harbor CRISPR-Cas systems, the prokaryotic adaptive immune systems capable of DNA-encoded, RNA-guided nucleic acid degradation that typically serves as antiviral defenses (5, 6). Lactobacilli are slightly enriched for CRISPR-Cas systems, as about 40% of bacteria and, as estimated by a previous study, approximately 46% of lactobacilli possess at least one CRISPR-Cas system (7). Currently, CRISPR-Cas systems of lactobacilli comprise both DNA-targeting and RNA-targeting systems of types I, II, and III (6). Type II CRISPR-Cas systems are RNA guided and DNA targeting and utilize a single effector protein, Cas9, to produce a double-strand break at a target site (8). In contrast, type I systems use a multi-protein Cascade complex for RNA-guided DNA binding at the target site, followed by recruitment of the Cas3 exonuclease for processive DNA cleavage (9). Type III systems, similarly, use the multi-protein Csm complex for RNA-guided RNA targeting, with subsequent ssDNA cleavage and further activities imparted by secondary messenger molecules (10). Altogether, these systems provide a diverse suite of CRISPR-Cas-mediated DNA- and RNA-targeting and degradation mechanisms. CRISPR-Cas systems would most typically use these mechanisms to defend against invading phage, and recent work predicted an abundance of diverse prophage (integrated lysogenic phage) sequences within lactobacilli (11, 12). Many *L. salivarius* strains were predicted to have multiple intact prophages alongside CRISPR-Cas systems, and some of these systems possessed self-targeting spacers targeting prophages within the same host (11). This dynamic between CRISPR-Cas systems and prophages has been documented, and prophages are associated with CRISPR-Cas autoimmunity (13). As CRISPR-Cas systems are capable of lethal nucleic acid degradation, bacteria circumvent self-targeting through various mechanisms. However, autoimmunity through CRISPR-Cas system deterioration is relatively rare (13).

Surprisingly, relatively little research has been conducted on the activity of CRISPR-Cas systems of *Ligilactobacillus*, particularly the most abundant member of the group, *L. salivarius*. Furthermore, there is a lack of functional characterization of *L. salivarius* CRISPR-Cas systems and how they may interact with coexisting prophage elements. Here, we performed phylogenetic comparisons of over 500 publicly available *Ligilactobacillus* genomes and predicted CRISPR-Cas system occurrence. We then focused on two *L. salivarius* strains, NCK 1352 and NCK 1355, with type I, II, and III CRISPR-Cas systems among them, and examined CRISPR-Cas system functionality in their native hosts. Following the prediction of CRISPR-Cas elements essential for interference activity, such as the CRISPR RNA (crRNA) and the trans-activating crRNA (tracrRNA), we tested the effector proteins and complexes of these CRISPR-Cas systems in a cell-free assay to examine functionality in a cell-free, exogenous context. We paired RNA sequencing with mitomycin C treatment to validate prophage predictions and confirm their activity to better understand interactions between CRISPR-Cas systems and self-targeting spacers with prophage target sites. We demonstrate that *L. salivarius* CRISPR-Cas systems target inducible prophage elements and retain activity despite maintaining self-targeting spacers.

## RESULTS

### *Ligilactobacillus* phylogenetic analysis and CRISPR-Cas system prediction

**Fig 1:**
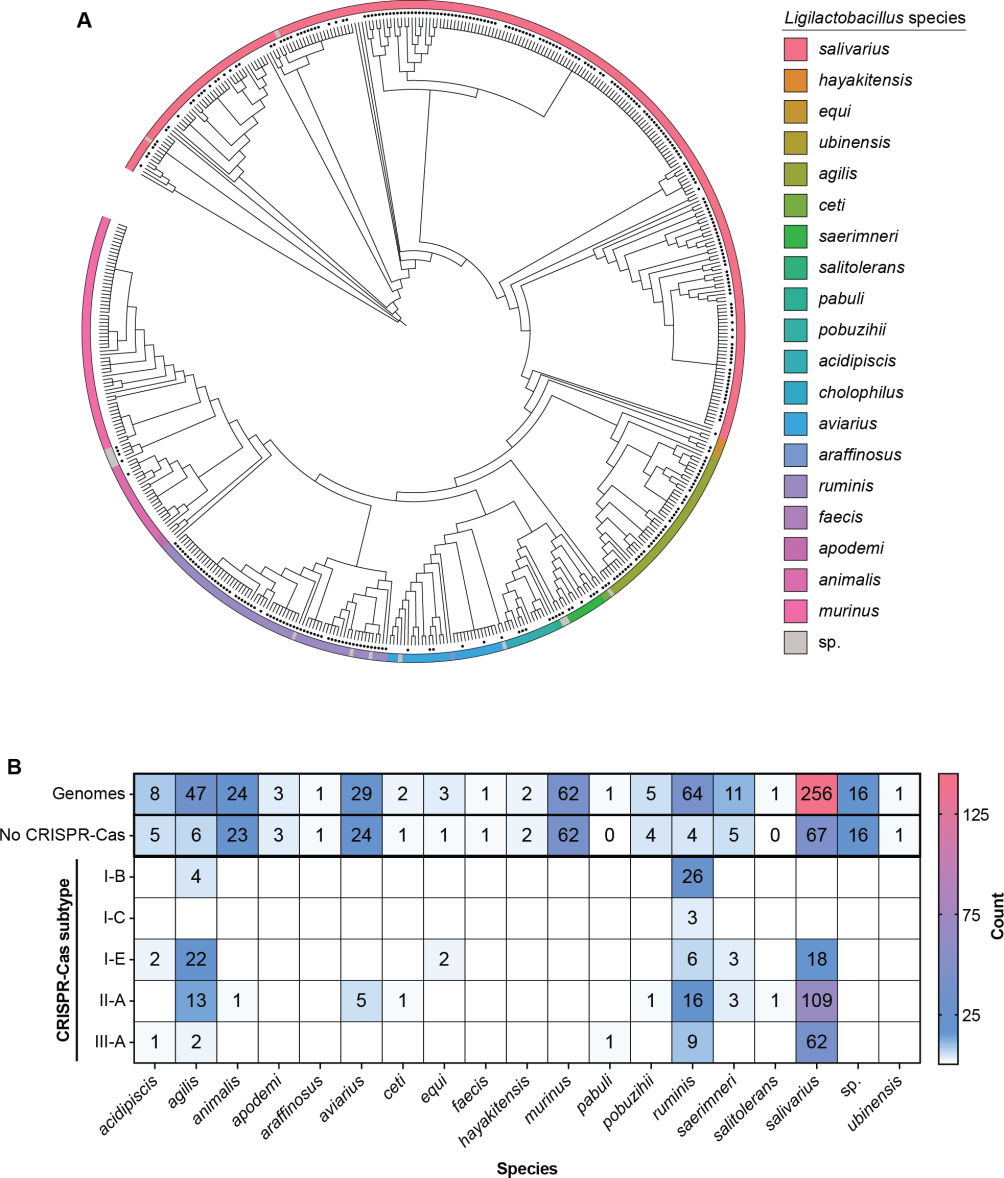
Phylogeny and CRISPR-Cas system diversity in the *Ligilactobacillus* genus. (**A**) Phylogenetic tree of *Ligilactobacillus* genomes based on ribosomal protein alignments. Black dots present at the end of a branch represent a predicted CRISPR-Cas system within the genome. (**B**) A heatmap depicting CRISPR-Cas system subtype abundance within species of the *Ligilactobacillus* genus. The total number of genomes represented for a species and the number of genomes without a predicted CRISPR-Cas system are also shown.

We conducted bioinformatic analyses to elucidate the phylogenetic relationships of species within the newly classified *Ligilactobacillus* genus, comparing the members based on ribosomal protein alignments. Notably, the genomes of *L. salivarius* dominate the NCBI RefSeq database, followed by other species such as *Ligilactobacillus ruminis* and *L. murinus* (Fig. 1A). This prevalence and over-abundance of specific members of the genus showcase clustering of genomes within these species, suggesting a possible exhaustive sampling from similar environmental niches or sources. Other members, such as *Ligilactobacillus araffinosus* and *Ligilactobacillus salitolerans*, are arguably underrepresented or undersampled.

Further analysis involved predicting complete CRISPR-Cas systems across the genus, categorized by subtype (Fig. 1B). On a subtype level, complete CRISPR-Cas systems were predicted within the *Ligilactobacillus* genus (Fig. 1B). From over 500 *Ligilactobacillus* genomes, this analysis revealed a diverse set of CRISPR-Cas systems, encompassing both classes and including three types and five subtypes: I-B, I-C, I-E, II-A, and III-A. Expectedly, species representation did not correlate with the presence of CRISPR-Cas content. For instance, the 62 genomes of the relatively well-represented *Ligilactobacillus murinus* lack any CRISPR-Cas systems, whereas 6 out of the 11 genomes of the less-represented *Ligilactobacillus saerimneri* contain complete CRISPR-Cas systems. The most abundant member of the genus, *L. salivarius*, primarily possesses type II-A, III-A, and I-E systems, with 109 type II-A systems being more prevalent than the 62 type III-A and 18 type I-E systems. Type I-C systems were found to be rare, exclusively present in *Ligilactobacillus ruminus* genomes. Also, *L. ruminus* is the only species with all CRISPR-Cas system subtypes identified in the genus. The *Ligilactobacillus agilis* CRISPR-Cas content is almost as diverse, representing four subtypes but lacking type I-C systems. However, some species, such as *Ligilactobacillus faecis* and *Ligilactobacillus ubinensis*, had no predicted CRISPR-Cas systems, though we hesitate to claim these species are generally lacking in CRISPR-Cas systems due to underrepresentation within the data set. We decided to focus our subsequent analyses on *L. salivarius* given the high representation within the genus and the presence of three different types of CRISPR-Cas systems, types I-E, II-A, and III-A, within these genomes.

### Comparative analyses of *L. salivarius* CRISPR-Cas systems

**Fig 2:**
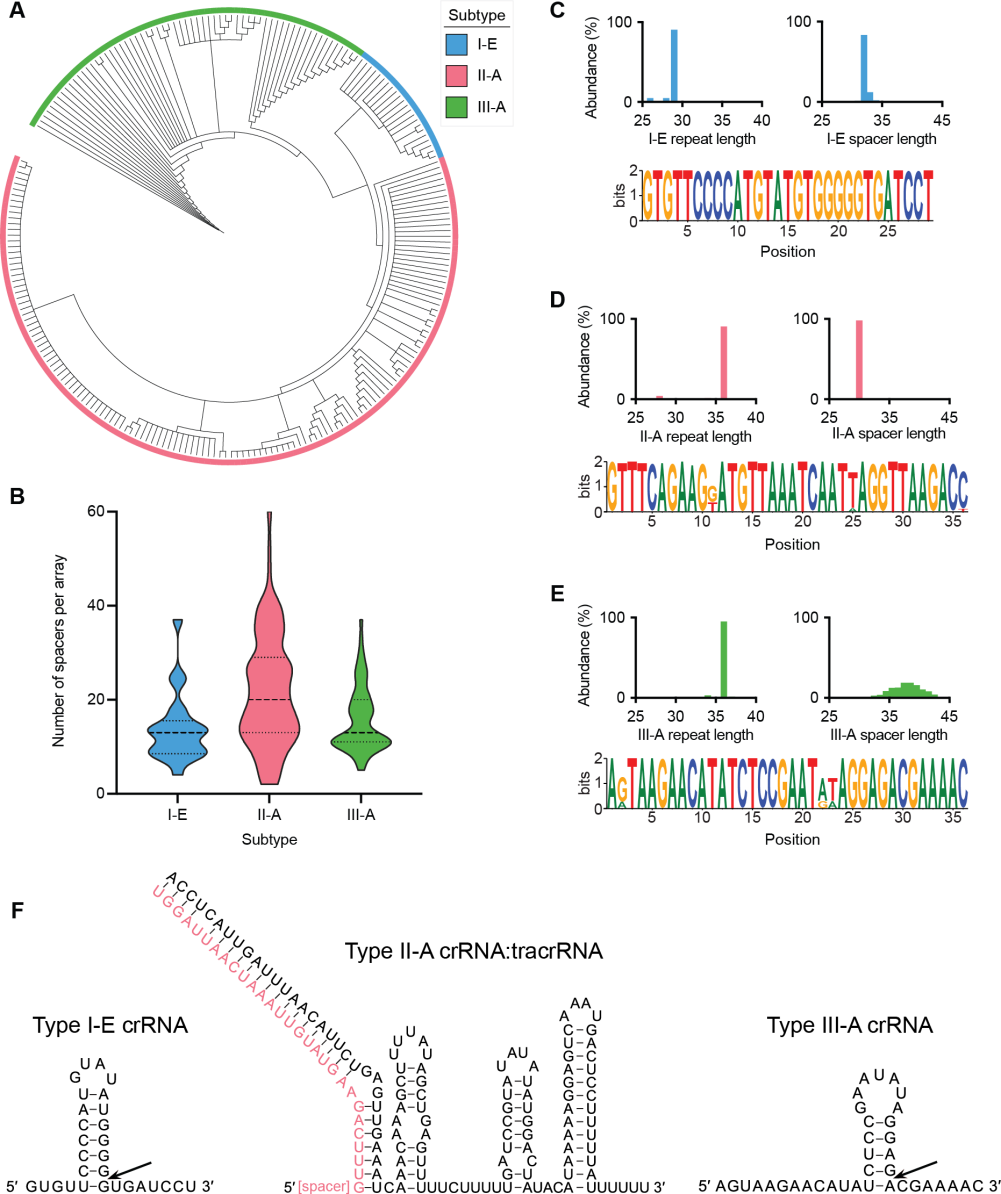
Comparative analyses of *Ligilactobacillus salivarius* CRISPR-Cas systems. (**A**) Phylogenetic tree based on an alignment of Cas1 proteins from type I-E, II-A, and III-A CRISPR-Cas systems of *L. salivarius*. (**B**) Abundance data for the number of spacers per array per CRISPR-Cas subtype. For each data set, the dotted lines represent the first quartile and third quartile, and the dashed line represents the median of the data distribution. (**C**) Abundance data for type I-E repeat and spacer lengths and a WebLogo of the type I-E repeat sequence. (**D**) Abundance data for type II-A repeat and spacer lengths and a WebLogo of the type II-A repeat sequence. (**E**) Abundance data for type II-A repeat and spacer lengths and a WebLogo of the type III-A repeat sequence. (**F**) Predicted secondary structures of the type I-E crRNA, type II-A crRNA:tracrRNA duplex, and type III-A crRNA. The crRNA processing points are noted with arrows.

First, as a basis for comparison, we aligned the Cas1 proteins derived from the *L. salivarius* CRISPR-Cas systems (Fig. 2A). Though not necessarily found in all CRISPR-Cas loci, Cas1 is a component of the acquisition stage of CRISPR-Cas adaptive immunity and is generally present within the type I-E, II-A, and III-A CRISPR-Cas systems found within *L. salivarius* (14). Expectedly, the phylogeny of these Cas1 proteins reveals apparent conservation based on the CRISPR-Cas subtype, an occurrence explained by the coevolution of the Cas1 proteins with the CRISPR-Cas system type (15). Additionally, multiple clusters of highly similar Cas1 proteins are seen within each subtype, with type II-A systems having relatively large clusters of similarity. Type I-E and type III-A Cas1 proteins in our data set showed the greatest dissimilarity, with only about 10% sequence identity. In comparison, type II-A Cas1 proteins were about 17% and 25% similar to Cas1 derived from type I-E and III-A systems, respectively. We next examined the CRISPR array length, based on the number of spacers per array, for each subtype (Fig. 2B). We found that type II-A systems not only possess the most diversity in CRISPR array length but also maintain the longest CRISPR arrays with upwards of 60 spacers in a single array. In contrast, type II-A and type III-A CRISPR arrays were generally less than 20 spacers in length. Next, we determined the CRISPR repeat and spacer length distribution across subtypes (Fig. 2C through E). Repeat sequences and lengths for each subtype were generally consistent, with one size significantly represented. The most abundant repeat lengths were 29, 36, and 36 bp for type I-E, II-A, and III-A systems, respectively. In contrast, the CRISPR array spacer length was conserved within type I-E and II-A systems but very diverse within type III-A systems. The most abundant spacer lengths for type I-E and II-A systems were 32 and 30 bp, respectively. Type III-A spacers, however, broadly ranged from 30 to 44 bp in length. This observed lack of conservation in type III-A spacer length is congruent with past findings that show more relaxed spacer acquisition lengths for this subtype (16). Finally, we predicted the secondary structures of the type I-E and type III-A crRNA species and the duplex structure of the type II-A crRNA and tracrRNA molecules (Fig. 2F). The type I-E and type III-A crRNA secondary structures include an expected hairpin (stem-loop) structure involved in Cas6 processing and Cascade binding (17). By aligning the type II-A crRNA repeat sequence with the predicted tracrRNA, we identified an anti-repeat region of the tracrRNA. The duplex species possesses clear lower stem, bulge, upper stem, and hairpin structures found in many similar tracrRNA (6).

### CRISPR-Cas systems of *L. salivarius* strains NCK 1352 and NCK 1355

**Fig 3:**
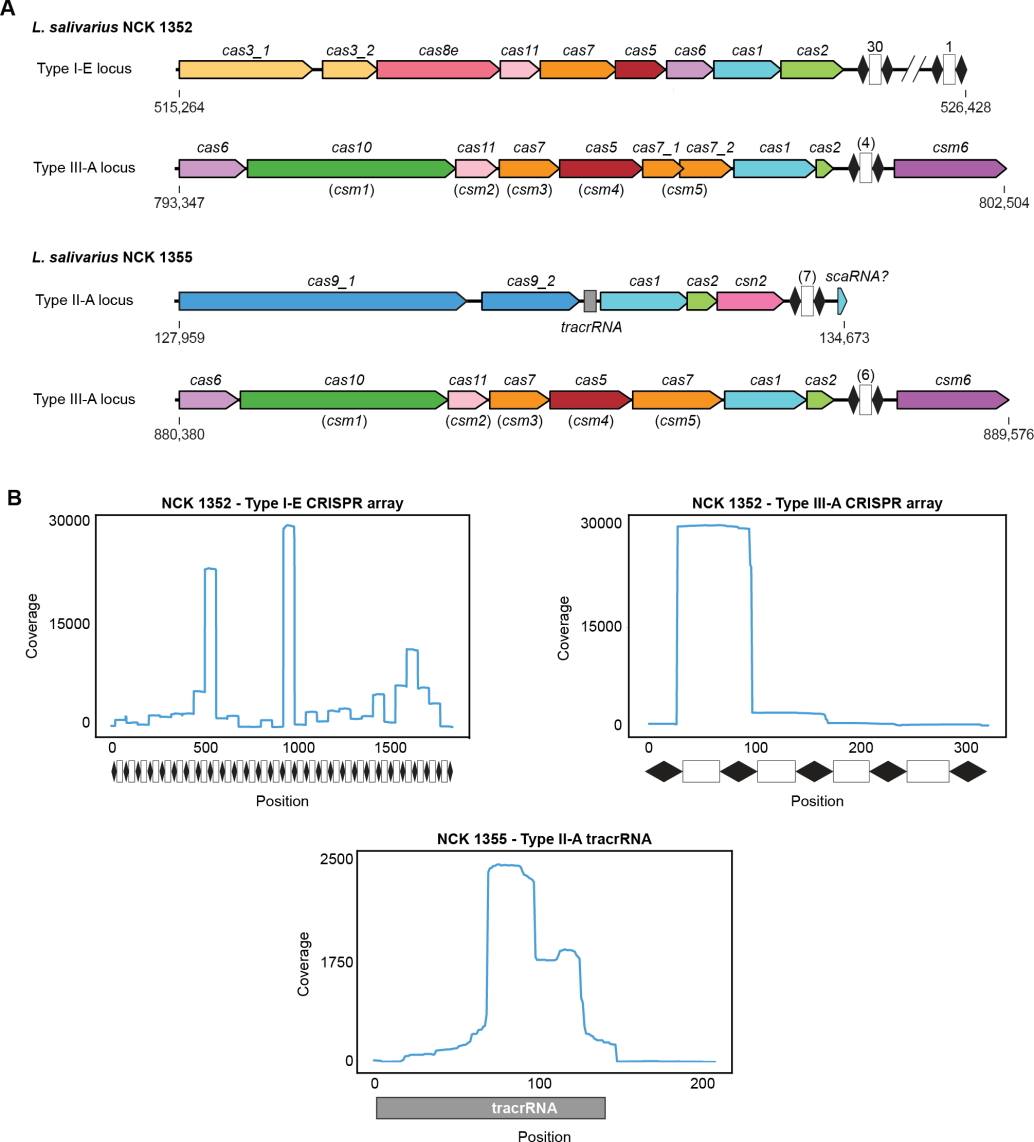
CRISPR-Cas systems of *L. salivarius* strains NCK 1352 and NCK 1355. (**A**) CRISPR-Cas loci within the genomes of *L. salivarius* strains NCK 1352 and NCK 1355. CRISPR arrays, depicted as black diamonds (repeats) flanking white rectangles (spacers), are shown with the total number of spacers in each array. (**B**) Small RNA coverage graphs for the CRISPR arrays and putative tracrRNA loci shown in **A**. The position information shows the length of each CRISPR array or tracrRNA. Transcription of the tracrRNA begins around position 150 and proceeds from right to left along the shaded tracrRNA region.

The CRISPR-Cas systems of two available *L. salivarius* human endoscopy sample isolate strains, NCK 1352 and NCK 1355, were further analyzed due to their intriguing CRISPR-Cas system diversity. We examined the type I-E and type III-A CRISPR-Cas loci of NCK 1352 and the type II-A and type III-A CRISPR-Cas loci of NCK 1355 (Fig. 3A). In all CRISPR-Cas loci of NCK 1352 and NCK 1355, the systems possess the expected acquisition module proteins, Cas1 and Cas2, including Csn2 for the type II-A system (15). The type I-E system of NCK 1352 contains a relatively sizable CRISPR array with 30 spacers, while the other systems have CRISPR arrays with seven or fewer spacers. The NCK 1352 type I-E system possesses all genes for the associated RNA-guided DNA-binding Cascade complex: *cas8e*, *cas11*, *cas7*, *cas5*, and *cas6*. The type I-E system also contains an unusual *cas3* gene split into two coding sequences, possibly resulting in a Cas3 protein without usual DNA cleavage activity. Similarly, the type III-A locus of NCK 1352 contains all genes necessary for the RNA-guided RNA-targeting Csm complex: *cas6*, *cas10*, *cas11*, *cas5*, and multiple copies of *cas7* (formerly *csm3* and *csm5*), as well as the CRISPR-associated gene *csm6* that encodes for a dimeric RNA endonuclease (18, 19). Similar to the type I-E system, the type III-A system of NCK 1352 possesses an unusual split *cas7* (*csm5*) gene with two overlapping coding sequences. As this Cas7 (Csm5) is involved in the maturation of type III-A crRNAs and RNA targeting, the split nature of the gene may result in a loss of some Csm complex functionality (20). However, the type III-A locus of NCK 1355 contains the same *cas* and *csm* genes with no split nor additional coding sequences. Like the CRISPR-Cas loci of NCK 1352, the NCK 1355 type II-A locus contains a split *cas9* gene with two distinct coding sequences. We also predicted a tracrRNA upstream of *cas1* and a potential small CRISPR-associated RNA immediately downstream of the CRISPR array (Fig. S1).

To further examine the functionality of the endogenous CRISPR-Cas systems within the *L. salivarius* NCK 1352 and NCK 1355, we performed RNA sequencing (RNA-Seq) on RNAs extracted from the strains. By mapping sequenced small RNAs back to the CRISPR loci, we found coverage graphs indicative of crRNA maturation for both the type I-E and type III-A systems of NCK 1352, suggesting that the Cas proteins involved in crRNA maturation are functional in both systems (Fig. 3B) (21). Like those previously studied, the type I-E CRISPR array peaks indicate preferential processing of specific crRNAs, likely due to the dynamic secondary structure of the pre-crRNA transcript (22, 23). The coverage profile for the type III-A CRISPR array shows a pattern where the most recently acquired spacer, located closest to the leader sequence driving CRISPR array transcription, is the most highly expressed crRNA with maturation frequency decreasing with the remaining spacers of the CRISPR array (6). The NCK 1355 type II-A tracrRNA coverage profile shows two prominent peaks, suggesting that there may be a longer 109-nt tracrRNA species and a shorter 52-nt species that may not contain the final hairpin of the tracrRNA secondary structure shown in Fig. 2F. Interestingly, few small RNA reads mapped to the NCK 1355 type II-A and type III-A CRISPR arrays and no discernable crRNA maturation patterns were present, suggesting that critical enzymes involved in the crRNA maturation process may be nonfunctional or inhibited by other proteins, such as anti-CRISPRs (24).

### Cell-free characterization of *L. salivarius* CRISPR-Cas systems

**Fig 4:**
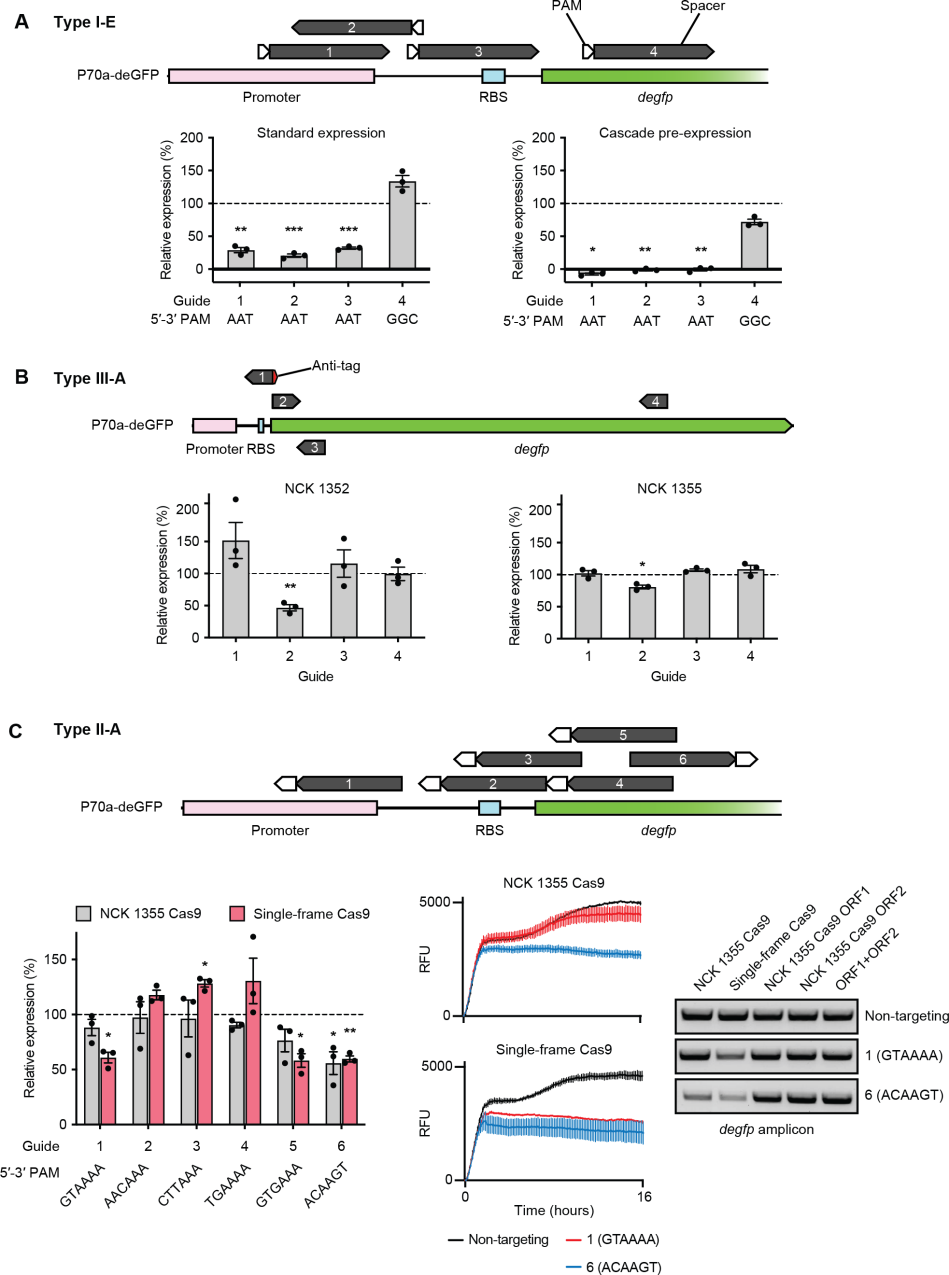
Cell-free functional characterization of *L. salivarius* NCK 1352 and NCK 1355 CRISPR-Cas systems. (**A**) A partial plasmid map of P70a-deGFP is shown with experimental PAM and target site positions. Relative GFP expression is shown for experiments with standard expression and pre-expression. (**B**) A partial plasmid map of P70a-deGFP is shown with experimental target sites. Spacers above the line target the coding strand sequence, while the spacer below the line targets the template strand sequence. Relative GFP expression is shown based on targeting by the Csm complex derived from strain NCK 1352 or NCK 1355. (**C**) A partial plasmid map of P70a-deGFP is shown with experimental PAM and target site positions. Relative GFP expression is shown for experiments with the NCK 1355-derived *cas9* genes or the single-frame *L. salivarius cas9* gene. Fluorescence over time data for three guides of interest are shown with associated *degfp* PCR amplicons. GFP fluorescence data are the average with SD of endpoint relative expression compared with a nontargeting control from *n* = 3 experiments. In the bar charts, dotted horizontal lines at *y* = 100% represent the expression of the nontargeting control reaction. Statistical significance is shown as *P ≤ 0.05, **P ≤ 0.01, and ***P ≤ 0.001.

Based on the unusual genetic makeup of the CRISPR-Cas loci and insights into endogenous system functionality, we decided to exogenously test the four CRISPR-Cas systems from *L. salivarius* NCK 1352 and NCK 1355 for further functional characterization. To test our systems outside their native context, we took advantage of recent advances in cell-free transcription-translation assays, especially for testing CRISPR-Cas systems (25). First, we tested the DNA binding activity of the NCK 1352 type I-E Cascade complex in the absence of Cas3 and its associated DNA cleavage activity. We developed expression vectors to express a minimal (single-spacer) CRISPR array and the Cascade complex and designed spacers to target various sites within the regulatory and coding sequence of a GFP gene (*degfp*) of a reporter plasmid (Fig. 4A). We primarily chose target sites with a 5′ end-proximal putative functional PAM (5′-AAT-3′) predicted in previous work (26). Another target site possessed a predicted nonfunctional PAM (5′-GGC-3′) to serve as a comparison for PAM specificity (guide #4). We observed visible repression, presumably due to DNA binding activity, in two assay setups by monitoring fluorescence relative to a nontargeting control spacer. In the standard expression scheme, where the expression and reporter vectors are added to the reaction concurrently, all reactions involving the targeting of a target site with a 5′-AAT-3′ PAM resulted in approximately 25% relative expression of the GFP reporter, while targeting a site with a 5′-GGC-3′ PAM resulted in reporter expression comparable to that of the nontargeting control. We also tested a pre-expression scheme often performed when testing CRISPR-Cas systems in transcription-translation assays, where the CRISPR and Cas components are expressed for a period before the reporter plasmid is added to the reaction (25). By pre-expressing the CRISPR and Cascade components from our expression vector, we observed striking repression from reactions with our predicted functional 5′-AAT-3′ PAM, all below the limit of detection. Interestingly, the reaction with our predicted nonfunctional 5′-GGC-3′ PAM displayed about 75% relative expression to the control, suggesting that overexpression of Cascade complexes in this context may result in PAM promiscuity or enough interaction with the target site possessing a less preferential PAM to elicit repression. Though PAM recognition initiates the DNA binding activity of Cascade, our spacers were designed with perfect complementarity to our chosen target sites, which likely impacted DNA binding activity with target sites possessing PAMs of lower affinity to our Cascade complex in this context of overexpression (27). These results show that the type I-E Cascade complex can bind DNA when expressed in an exogenous setting.

We next tested the RNA-targeting type III-A systems of both strains with a similar expression setup to that of the type I-E system, where the CRISPR, Cas6, and Csm complex (Cas10-Cas11-Cas7-Cas5-Cas7) components were expressed, and spacers were primarily designed to target different locations within the *degfp* transcript (Fig. 4A). Of our four target sites, three were designed to target the RNA sequence of the *degfp* transcript, while one (guide #3) was designed to target the template strand as a control for RNA specificity. In addition, one of the transcript target sites (guide #1) possesses an adjacent sequence (5′-CUUUUC-3′) with near-perfect complementarity to the 3′ end of the type III-A CRISPR repeat sequence (5′-GAAAAC-3′). This sequence is known as an anti-tag and, like the PAM of other CRISPR-Cas systems, allows for self versus nonself discrimination by the Csm complex (28). Though the Csm complex can still cleave RNA molecules possessing the anti-tag sequence adjacent to a target site, the ssDNA cleavage activity of Cas10 is not initiated. For the type III-A system of NCK 1352, we saw evident repression only at the target site relatively early in the *degfp* transcript without an anti-tag (guide #2), while GFP expression in other reactions was comparable to that of the control. A similar pattern is seen for the type III-A system of NCK 1355, although the extent of repression seen for guide #2 is less than that produced by the NCK 1352 type III-A system. Though we repeated these reactions by first pre-expressing the CRISPR and Cas components, no apparent repression was observed in that context for either system (Fig. S2A). These findings suggest these type III-A systems can cleave RNA and potentially have ssDNA activity. However, the lack of repression seen by targeting relatively late in the *degfp* transcript indicates that the target site location may be material for achieving repression in this context.

The type II-A system of NCK 1355 was tested by expressing the two Cas9 genes (*cas9_1*, *cas9_2* shown in Fig. 3A) from an expression vector (“NCK 1355 Cas9”) while expressing an engineered single-guide RNA (sgRNA) from a linear expression construct (Fig. 4C). We designed spacers targeting sites in the upstream regulatory and early coding sequence of *degfp*, primarily with predicted 3′-proximal, functional PAMs of 5′-NNaWNW-3′ from previous work (29). In addition to testing the native *cas9* genes of NCK 1355, we also tested a “single-frame” *cas9* gene found within other *L. salivarius* strains for which a single adenine insertion within the *cas9_1* gene of NCK 1355 restores a single-frame, full-length coding sequence for Cas9. Repression activity was somewhat similar between the two Cas9s tested, where guides #5 and #6 targeting early in the *degfp* gene, with 5′-GTGAAA-3′ and 5′-ACAAGT-3′ PAMs, resulted in noticeable repression. Targeting with guide #1, which targets a promoter sequence with a 5′-GTAAAA-3′ PAM, showed approximately 55% relative repression for the single-frame Cas9 but poor repression for the NCK 1355 Cas9. Other target sites (guides #2–4) showed no apparent repression activity for either Cas9 tested. We decided to further analyze the DNA binding and cleavage activity of these Cas9 by PCR amplifying a ~1-kb region, encompassing all the target sites of the reporter plasmid, from the DNA present at the end of the assay reactions. We also show PCR products amplified from similar experiments in which we individually expressed the NCK 1355 *cas9_1* and *cas9_2* genes on separate plasmids, in individual reactions or together in a single reaction. However, no apparent repression was seen in these experiments (Fig. S2B). Interestingly, our PCR products showed intensity correlated with the repression activity of the corresponding assay reactions. In contrast, our nontargeting reaction showed similar amplification in all scenarios. The single-frame Cas9 guide #1 reaction showed slightly less amplification than the control, while both Cas9s tested showed less amplification for guide #6 relative to the controls. Altogether, these results suggest that both the NCK 1355 Cas9 split between *cas9_1* and *cas9_2* and the single-frame Cas9 may, to some extent, possess functional DNA binding and cleavage activity.

### *L. salivarius* self-targeting spacers and their targets

**Fig 5:**
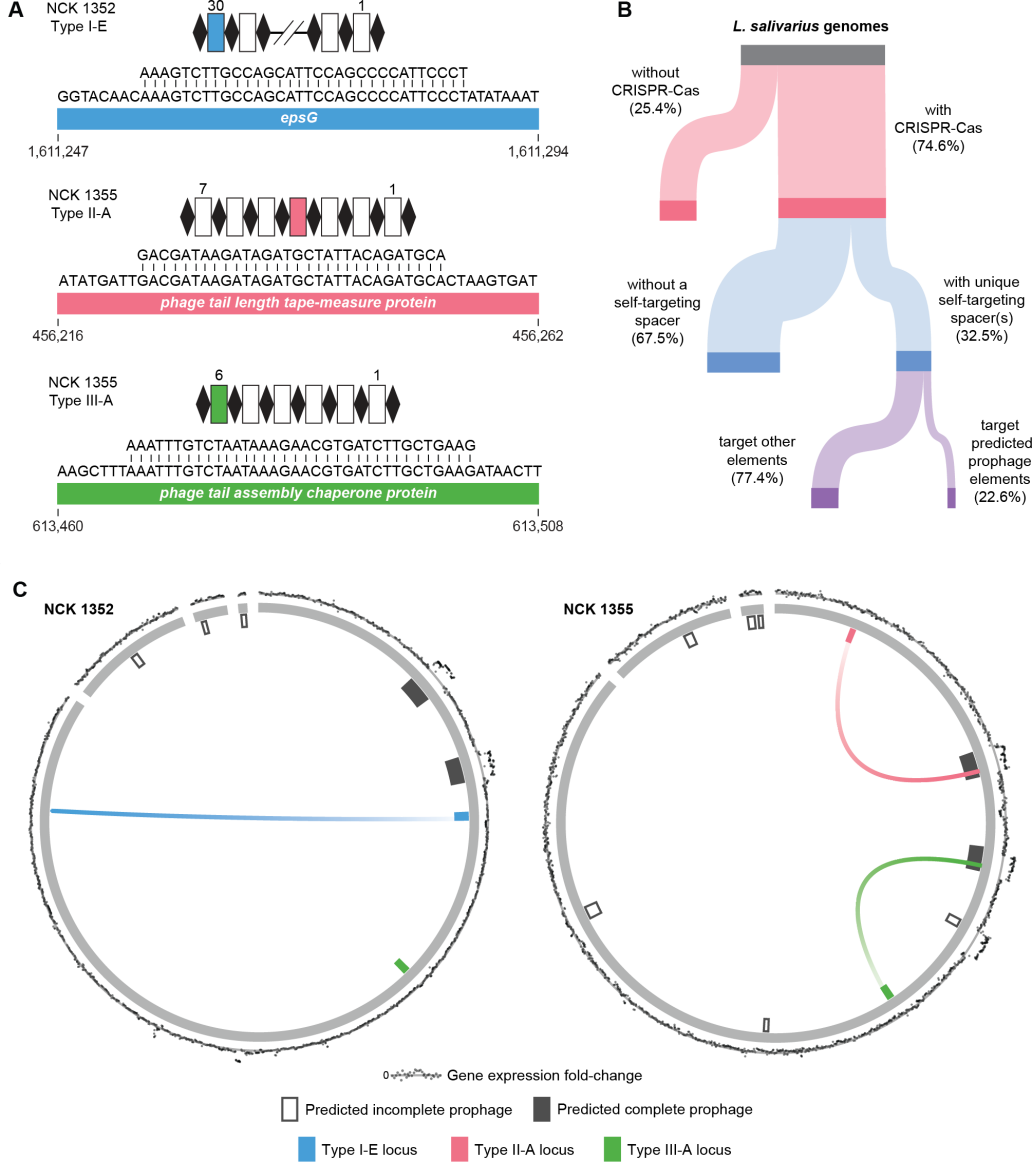
Self-targeting CRISPR spacers and prophage targeting in *L. salivarius* strains. (**A**) Self-targeting spacers of strains NCK 1352 and NCK 1355. The associated CRISPR-Cas subtype and genomic coordinates are shown for each spacer-target alignment. CRISPR arrays are depicted as black diamonds (repeats) flanking white rectangles (spacers), and the self-targeting spacer is colored within each CRISPR array. Gene names or gene products are shown. (**B**) A Sankey diagram depicting the frequency of *L. salivarius* CRISPR-Cas systems, self-targeting spacers, and elements targeted by self-targeting spacers. (**C**) Circos plots depicting the genomes of strains NCK 1352 and NCK 1355. The middle gray segments correspond to chromosome and plasmid sequences, while inner segments show predicted prophage and CRISPR-Cas regions. Self-targeting spacer target sites are displayed as colored lines from the associated CRISPR-Cas system to the target site. The outer plot ring depicts RNA-Seq differential gene expression fold change data for genes at the same position in the genome, with higher fold-change data farthest from the line.

Given the multiple split *cas* genes within the diverse CRISPR-Cas systems of the *L. salivarius* NCK 1352 and NCK 1355 strains, we investigated a potential explanation. The presence of self-targeting spacers, which target genomic loci within the host organism’s genome that harbors the CRISPR-Cas system, often indicates nonfunctional or inhibited CRISPR-Cas systems (24). Self-targeting spacers may coincide with mutations within *cas* genes, such as premature stop codons leading to truncated proteins (24). Interestingly, we identified three unique self-targeting spacers across the three types of CRISPR-Cas systems of *L. salivarius* strains NCK 1352 and NCK 1355 (Fig. 5A). The type I-E CRISPR-Cas system of NCK 1352 possesses a self-targeting spacer that targets the *epsG* gene at a site with a 5′-AAC-3′ PAM. The NCK 1355 self-targeting spacers, however, both target prophage genes: the type II-A target site with 5′-CTAAGT-3′ PAM is a phage tail-length tape-measure protein gene, while the type III-A target site is a phage tail assembly chaperone protein. Due to this surprising rate of self-targeting spacers within just two strains, we extended our search to all the *L. salivarius* genomes (Fig. 5B). We discovered that, in approximately 75% of *L. salivarius* genomes that possess complete CRISPR-Cas systems, unique self-targeting spacers are spread across roughly a third (32.5%) of the genomes. Of these unique self-targeting spacers, approximately one-quarter (22.5%) are predicted to target predicted prophage elements, while the remaining portion (77.4%) targets other elements (Table S3). In these *L. salivarius* genomes, no significant association was detected between the presence of CRISPR-Cas systems and the number of predicted prophages within a genome. As anti-CRISPR proteins are commonly associated with self-targeting spacers, we conducted a preliminary search for anti-CRISPR proteins within the *L. salivarius* genomes that contain self-targeting spacers (30). We found a variety of potential anti-CRISPR proteins with similarity to both verified and predicted anti-CRISPRs from the literature, with most of the hits being associated with anti-CRISPRs that inhibit type II systems (Table S5).

Our NCK 1352 and NCK 1355 strains, each with at least one self-targeting spacer and predicted prophage elements, prompted further investigation into prophage activity in these strains. We sequenced RNA from these strains under untreated and mitomycin C-treated conditions and performed differential gene expression analysis (Table S6). Mitomycin C, an agent known to disrupt DNA replication mechanisms, is commonly used to induce prophages from lysogenic to lytic states (31). The comparative gene expression profiles from untreated versus treated samples aimed to ascertain the inducibility of these prophage elements and their potential role in the interactions between CRISPR-Cas and mobile genetic elements. Intriguingly, we found that both strains’ predicted complete prophage elements were highly upregulated post-mitomycin C exposure (Fig. 5C). Additionally, a predicted incomplete prophage element in NCK 1355 also showed apparent upregulation, demonstrating the effectiveness of such data for identifying the completeness and genomic boundaries of such mobile genetic elements. We also show the self-targeting spacer target sites stemming from their associated CRISPR-Cas system, highlighting that these targeted elements are likely active and may have influenced the existing state of the coexisting CRISPR-Cas systems.

## DISCUSSION

Lactobacilli, renowned as industrial workhorses and useful probiotics, have historically been insightful for the research of CRISPR-Cas systems within their native context (14). We show the diversity of the recently reclassified *Ligilactobacillus* genus and the breadth and depth of representation within publicly available genomes dominated by *L. salivarius* (1). Our findings also revealed three types of CRISPR-Cas systems within the genus, including type III-A systems. Notably, type III systems are a relative rarity among lactobacilli, though they occur in *Streptococcus* and are absent within other lactic acid bacteria such as *Bifidobacterium* (6, 32, 33). Interestingly, we found that CRISPR-Cas diversity is quite variable within species of *Ligilactobacillus*, with species such as *L. ruminis* containing all predicted CRISPR-Cas subtypes of the genus, while *L. salivarius* has all types I, II, and III, thus including both DNA-targeting and RNA-targeting systems and a wide range of anti-viral mechanisms.

Our findings regarding the CRISPR content within these *L. salivarius* systems were generally typical. CRISPR repeat and spacer lengths were broadly consistent within each type, except type III-A spacer lengths deviating in a manner observed previously (16). The secondary structures of the associated crRNAs were also typical, with expected hairpins involved in Cas6 recognition and processing. The type II-A tracrRNA, however, had an unusual form of three hairpins when compared with the predicted tracrRNAs of other lactobacilli (6), though our RNA sequencing data suggest that there may be a shorter tracrRNA species that lacks the final hairpin (Fig. 3B). Overall, the RNA sequencing data indicate that the type I-E and type III-A systems of NCK 1352 are, at a minimum, capable of crRNA maturation in a native context. The lack of small RNA coverage at the NCK 1355 type II-A and type III-A CRISPR loci, despite observing coverage in these regions from total RNA sequencing, indicates that there may be a lack or inhibition of expected function for crRNA maturation for these systems in this context, therefore limiting the ability of these systems to actively bind or cleave nucleic acids in an RNA-guided manner. Intriguingly, the presence of a mature tracrRNA and no mature crRNAs provides a fascinating insight into a potential step in crRNA biogenesis that may be inhibited, such as by an anti-CRISPR, or absent due to poor or no facilitation by the endogenous Cas9 (34). Indeed, the Cas9 protein comprises multiple genes in NCK 1355 and is therefore potentially dimeric, which may explain the lack of crRNA maturation.

Despite the lack of evidence for a fully functional Cas9 protein, we decided to still test the NCK 1355 Cas9 alongside a representative single-frame *L. salivarius* Cas9 in a cell-free context, aiming to test the functionality of these proteins outside of the context of endogenous inhibitors that may be present in the strain. Likewise, to control for crRNA maturation variability, we engineered a sgRNA by fusing the NCK 1355 crRNA and tracrRNA into a sgRNA with a tetraloop linker sequence, as previously described (35). Our results suggest that both Cas9 proteins may be functional, though the single-frame Cas9 provided more substantial evidence for DNA cleavage activity across multiple target sites. The sgRNA structure used here may be broadly applicable but inefficient with these particular Cas9s (36). Alternatively, the transcription-translation assay incubation temperature of 29°C may not facilitate high activity for these proteins, or the predicted PAM sequences may require experimental confirmation using similar methods (37). However, more defined biochemical assays can further elucidate the activity of these Cas9 to validate their functionality confidently.

Our *in vitro* testing of the type I-E system revealed apparent DNA binding activity on a PAM-specific basis, confirming the prediction of the 5′-AAT-3′ PAM and the activity of the Cascade complex. However, we did not test the DNA cleavage of the Cas3 (split as *cas3_1* and *cas3_2* genes) associated with the type I-E system of NCK 1352. Similarly, though our results suggest activity for both type III-A systems, we did not test the activity of the associated Csm6 proteins nor differentiate GFP repression that resulted from RNA cleavage or Cas10-mediated DNA cleavage. Interestingly, the NCK 1352 system possesses a split Cas7/Csm5 (*cas7_1* and *cas7_2*; Fig. 3A) but exhibited stronger repression than the NCK 1355 type III-A system. These results and the lack of evidence for crRNA maturation for the NCK 1355 system suggest that there may be intrinsic differences in activity between the two complexes and Cas6 proteins. Unlike with the type I-E systems, pre-expression of the type III-A components resulted in no evident repression (Fig. S2A), suggesting that testing for type III-A systems may require further optimization or that pre-expression strategies are broadly, but not universally, applicable. Previous work involving testing a type III-A system of *Streptococcus thermophilus* in a transcription-translation assay expressed the Csm complex without Cas6 and instead expressed a mature crRNA species for targeting (38). It is possible that such alterations would result in improved repression activity for these *L. salivarius*-derived type III-A systems.

The presence of noncanonical CRISPR-Cas loci led us to examine the occurrence of self-targeting spacers within *L. salivarius* and, to our surprise, we discovered self-targeting spacers with perfect complementarity to their target sites among each of the three CRISPR-Cas system types in the two strains NCK 1352 and NCK 1355. We identified potential causes of the lack of function of the regular activity of these systems based on RNA sequencing and *cas* locus analyses; however, mutations or deletions in the CRISPR array can disrupt self-targeting (39). Further examination of the CRISPR repeats associated with the self-targeting spacers revealed that mutations within the CRISPR elements are potentially, but unlikely alone, involved in functional inhibition here. For example, the two repeats flanking the NCK 1352 type I-E self-targeting spacer each possess two mutations, relative to the consensus repeat, at the 3′ ends of the repeats. Meanwhile, the NCK 1355 type II-A self-targeting spacer has an upstream repeat with a single mutation situated in the upper stem of the crRNA:tracrRNA duplex, while the type III-A self-targeting spacer has no associated repeat mutations. This reveals that, unlike in some scenarios of self-targeting, CRISPR repeat mutations that result in poor crRNA maturation or binding are likely not the main factor in CRISPR-Cas system inhibition or escape of targeting in these strains.

Interestingly, we established that the NCK 1352 type I-E system can mature crRNA in an endogenous setting and that the Cascade complex has DNA-binding activity in an exogenous context. Though the 5′-AAC-3′ PAM of the type I-E self-target site may result in some DNA binding activity, as it is only a single base away from the validated 5′-AAT-3′ PAM, we did not observe any apparent downregulation of *epsG* in our sequencing data. It is tempting to speculate whether there is condition-dependent autoregulation through self-targeting of *epsG*, thus controlling the production of exopolysaccharide, as CRISPR-Cas systems have previously been implicated in the regulation of exopolysaccharide production in other bacteria (40). Meanwhile, the NCK 1355 type III-A self-target site does not possess an anti-tag sequence, and the type II-A self-target site has a 5′-ATAACT-3′ PAM, similar to the predicted PAM of 5′-NNaWNW-3′ (29). Interestingly, we predicted that most self-targeting spacers of *L. salivarius* do not target prophage elements. Further analysis of the PAMs associated with prophage and nonprophage self-target sites did not reveal any striking motifs or correlations, except a strong 5′-AAY-3′ PAM associated with three nonprophage self-target sites by type I-E systems (Fig. S3). Although we showed that the prophages within NCK 1352 and NCK 1355 are inducible, we did not establish whether these CRISPR-Cas systems play a defined role in actively targeting or regulating these mobile genetic elements. Our preliminary search for anti-CRISPR proteins did find potential type II-A anti-CRISPRs, and more undiscovered anti-CRISPRs may be present and acting on the type I-E and type III-A systems within these genomes. These strains likely contain a combination of potential responses to self-targeting spacer acquisition, such as mutations in CRISPR repeats, *cas* genes, or PAMs, and the presence of anti-CRISPR proteins. Regardless of the escape mechanism, the CRISPR-Cas systems of these *L. salivarius* strains retain some degree of activity within our *in vitro* testing context. We believe these findings will contribute to the future characterization of these CRISPR-Cas systems, the development of these CRISPR-Cas systems as genome editing tools, and elucidating interactions between these systems and coexisting prophages.

## MATERIALS AND METHODS

### Phylogenetic tree construction

*Ligilactobacillus* genomes were downloaded from the National Center for Biotechnology Information (NCBI) RefSeq database. A phylogenetic tree based on ribosomal proteins was constructed as previously described (41). For each genome, genes were predicted using Prodigal (42) and searched using HMMER3 (43) with HMM models for 16 ribosomal proteins of interest. For each set of identified ribosomal marker proteins, individual alignments were performed using MUSCLE (44), followed by trimming of alignments with trimAl (45) and concatenation of the ribosomal protein alignments using BinSanity (46). A phylogenetic tree of this alignment was generated using FastTree (47), and this phylogenetic tree was visualized using iTOL (48).

### CRISPR-Cas system analyses and visualization

CRISPR-Cas systems were predicted within *Ligilactobacillus* genomes from RefSeq using CRISPRCasTyper v1.8.0 (49) with flags --exact_stats, --searchWL 7, and --keep_tmp. CRISPR-Cas systems containing both a detected CRISPR array and *cas* genes required for interference activity were included in downstream analyses. Cas1 proteins from *L. salivarius* CRISPR-Cas systems were extracted from the CRISPRCasTyper data files, aligned using MUSCLE, and visualized using FastTree. Also extracted from the resulting data files were the number of spacers per CRISPR array, repeat length, spacer length, and CRISPR repeat sequence for each CRISPR-Cas subtype. For each subtype, CRISPR repeat sequences were manually inspected for improper orientation, corrected if necessary, and then used to produce a WebLogo (50). A representative tracrRNA species was predicted according to known tracrRNA characteristics within lactobacilli (6). The selected tracrRNA sequence and the most abundant CRISPR repeats were used for tracrRNA and crRNA structure prediction with NUPACK (51). The CRISPR-Cas systems of *L. salivarius* strains NCK 1352 and NCK 1355 were visualized using the DNA Features Viewer Python library (52). CRISPRCasTyper results are in Table S1.

### Cell-free transcription-translation assays

Cell-free transcription-translation (TXTL) assays were performed using the myTXTL Linear DNA Expression Kit (Daicel Arbor Biosciences), and assay conditions were modeled after similar work (25). For the assays, each reaction mixture totaled 12 µL, comprising 9.375 µL of the master mix along with additional expression constructs. Subsequently, these mixtures were divided into two 5-µL technical replicates. Standard components across all reactions, at their final concentrations, included P70a-T7rnap (0.2 nM), pTXTL-P70a-deGFP (0.5 nM), and IPTG (0.5 mM). All Cas effector proteins were expressed from a plasmid, with a T7 promoter and IPTG-inducible lac operator, at a final concentration of 1 nM. This setup facilitated the expression of either a single or polycistronic transcript. Reactions investigating the type I-E Cascade complex included a single plasmid to express a single-spacer CRISPR array, *cas8e*, *cas11*, *cas7*, *cas5*, and *cas6*. Similarly, reactions testing the type III-A Csm complex included a single plasmid to express a single-spacer CRISPR array, *cas6*, *cas10*, *cas11*, *cas7* (*csm3*), *cas5*, and *cas7* (*csm5*). Assays testing Cas9 or its variants included a single plasmid to express *cas9* or individual ORFs. Each Cas9 reaction had a linear sgRNA expression construct containing a J23119 promoter, a 20-nt spacer, a sgRNA sequence, and a BBa_J61048 terminator. The sgRNA was designed based on a broadly applicable linker sequence between the crRNA anti-repeat and the tracrRNA, and the longer predicted tracrRNA species was used (36). Linear sgRNA constructs were ordered as gBlocks (IDT), resuspended in molecular-grade water, and used directly in reactions. Spacers were designed to guide targeting within the promoter or *degfp* gene of the pTXTL-P70a-deGFP plasmid to impact GFP fluorescence. Samples were loaded on a 96-well V-bottom plate (MilliporeSigma) and run for 16 hours at 29°C on a FLUOstar Omega microplate reader (BMG Labtech) while reading GFP fluorescence. Pre-expression experiments required an initial incubation phase, where the 12-µL reaction mixtures were placed in a microcentrifuge tube within a 29°C water bath for 3 hours before 96-well plate loading and plate reader measurements. Randomized, nontargeting spacer sequences served as a control for fluorescence repression comparisons, and all endpoint fluorescence values are relative to the endpoint value of the nontargeting spacer reaction. Background subtraction was performed for each experiment by subtracting the fluorescence of a master mix-only control from the fluorescence value of every experimental reaction. For each sample set, Welch’s *t*-test was performed to calculate significance.

### DNA manipulations

Genomic DNA was extracted from *L. salivarius* NCK 1352 and NCK 1355 with the DNeasy PowerLyzer Microbial Kit (QIAGEN). Expression plasmids for TXTL were created by PCR-amplifying the *cas* gene(s) from genomic DNA extracted from NCK 1352 and NCK 1355 and cloning the amplicons into the expression plasmid backbone (CloDF13 origin, *lacI*, and a T7 promoter with an IPTG-inducible lac operator) with the NEBuilder HiFi DNA Assembly Cloning Kit (NEB). CRISPR arrays and spacers were cloned into the expression plasmids using conventional restriction cloning and annealed oligos (IDT). Plasmids were cloned and propagated in NEB 10-beta cells (NEB) grown in LB broth and appropriate antibiotics. Plasmids were extracted using a Plasmid Midi Kit (QIAGEN) and were further purified using a Monarch PCR & DNA Cleanup Kit (NEB) before use in TXTL.

Completed TXTL reactions were diluted 1:10 with molecular-grade water and used as a template for PCR. PCR primers were designed to amplify a 926-bp region containing all type II-A spacer target sites tested on the pTXTL-P70a-deGFP plasmid. Each reaction was 50 µL in total and incorporated 25 µL Q5 Master Mix (NEB), 2.5 µL of each primer at 10 µM, 5 µL diluted TXTL reaction, and 10 µL water. Thermocycling conditions were an initial denaturation step of 30 seconds at 98°C, 25 cycles of [10 seconds at 98°C, 15 seconds at 68°C, and 20 seconds at 72°C], and a final extension step of 2 minutes at 72°C. PCR products were visualized on a 1% agarose gel with ethidium bromide. The primers used are in Table S2.

### Prediction of self-targeting spacers and prophages

Self-targeting spacers within *L. salivarius* genomes were predicted based on custom scripts from previous work (53), which functions as described here. First, the CRISPR Recognition Tool (54) identifies CRISPR arrays within the genome. Next, a BLASTN search is performed on the genome using identified spacers as a query (55). Mutations within the repeats flanking the self-targeting spacer are determined based on mismatches when aligned with the consensus repeat of the same CRISPR array. Mismatches between the self-targeting spacer and the target sequence are identified, and the upstream and downstream regions are extracted, providing putative PAM and anti-tag data. Upstream and downstream sequence data were used to generate WebLogos. Gene products of genes targeted by self-targeting spacers were identified when possible. Self-targeting spacer results are in Table S3.

Using *L. salivarius* genomes that contained self-targeting spacers, prophage regions were predicted using Phigaro (56). Self-targeting spacers were identified as prophage-targeting if the spacer targeted within a predicted prophage region or within 10 kb upstream or downstream from a predicted prophage region. Additionally, self-targeting spacers were identified as prophage targeting if a targeted gene was identified as prophage-related (e.g., “phage,” “tail,” or “terminase”) despite the gene not residing within or near a predicted prophage region. *L. salivarius* strains NCK 1352 and NCK 1355 were also analyzed using PHASTER for more information on complete and incomplete prophage regions (57). Phigaro results are presented in Table S4.

### Anti-CRISPR protein prediction

Verified and predicted Anti-CRISPR proteins were extracted from the Anti-CRISPRdb v2.2 database (30). The anti-CRISPR protein sequences were used as a query for a position-specific iterated BLAST (PSI-BLAST) search, with flags -qcov_hsp_perc 50 and -evalue 0.01, against a local database of *L. salivarius* proteins from genomes that each contain at least one self-targeting spacer. Anti-CRISPR PSI-BLAST results with associated Anti-CRISPRdb data are listed in Table S5.

### RNA-Seq preparation and analyses

The following methods were used for total RNA extraction of *L. salivarius* strains NCK 1352 and NCK 1352. Each strain was grown overnight in De Man-Rogosa-Sharpe (MRS; Difco) broth at 37°C without shaking, and this overnight culture was used for a 1.5% inoculum in 200 mL of pre-warmed MRS that was further cultured at 37°C without shaking. At a 600 nm optical density(OD_600nm_) value of approximately 0.25, each culture was split for untreated and mitomycin C-treated conditions. Immediately following culture splitting, mitomycin C (Sigma-Aldrich) was added to one culture at a final concentration of 0.75 µg/mL of culture. Untreated and treated cultures were further incubated at 37°C. When the OD_600nm_ of a culture reached approximately 0.7, cells were harvested by centrifugation for 10 minutes at 3,200 × *g* and 4°C, and cell pellets were flash frozen and stored at −80°C. We note that growth rates were delayed for mitomycin C-treated samples, but no severe reductions in OD were observed. According to manufacturer instructions, total RNA was extracted from cell pellets with the Zymo Direct-Zol RNA Miniprep Kit (Zymo Research). Library preparation and RNA sequencing for mRNA and small RNA were performed at the Roy J. Carver Biotechnology Center of the University of Illinois Urbana-Champaign. RNA-Seq data were mapped to NCK 1352 and NCK 1355 genomes using the Geneious read mapper of Geneious Prime 2023.1.1 (https://www.geneious.com) with default settings. Read coverage graphs were made using coverage data from the sequence alignment and map files exported from Geneious after read mapping. Differentially expressed genes between conditions were determined using DESeq2 (58), and the effect size estimate, as log2 fold change, was visualized on a Circos plot using Circa (http://omgenomics.com/circa). DESeq2 results are in Table S6. The results of genes within predicted prophage regions are indicated in this table.

## Data Availability

Following the analyses of this manuscript, the genomes of *L. salivarius* NCK 1352 and *L. salivarius* NCK 1355 were deposited to NCBI with genome accession numbers JBAGCZ000000000 and JBAGCY000000000. RNA-Seq datasets associated with *L. salivarius* NCK 1352 and *L. salivarius* NCK 1355 were deposited to NCBI under BioSamples SAMN39905277 and SAMN39905278. Data can be accessed at https://www.ncbi.nlm.nih.gov.

## SUPPLEMENTAL MATERIAL

Supplemental Figures msphere.00171-24-s0001.pdf Figures S1 to S3.

Supplemental Tables msphere.00171-24-s0002.xlsx Tables S1 to S6.

ASM does not own the copyrights to Supplemental Material that may be linked to, or accessed through, an article. The authors have granted ASM a non-exclusive, world-wide license to publish the Supplemental Material files. Please contact the corresponding author directly for reuse.
