# Supplementary material for: Characterization of Ligilactobacillus salivarius CRISPR-Cas systems
Source: mSphere. 2024 Jul 11;9(7):e00171-24. doi: 10.1128/msphere.00171-24 (PMC11288051; doi:10.1128/msphere.00171-24)
Supplement: Supplemental Figures — Figures S1 to S3. [file msphere.00171-24-s0001.pdf]

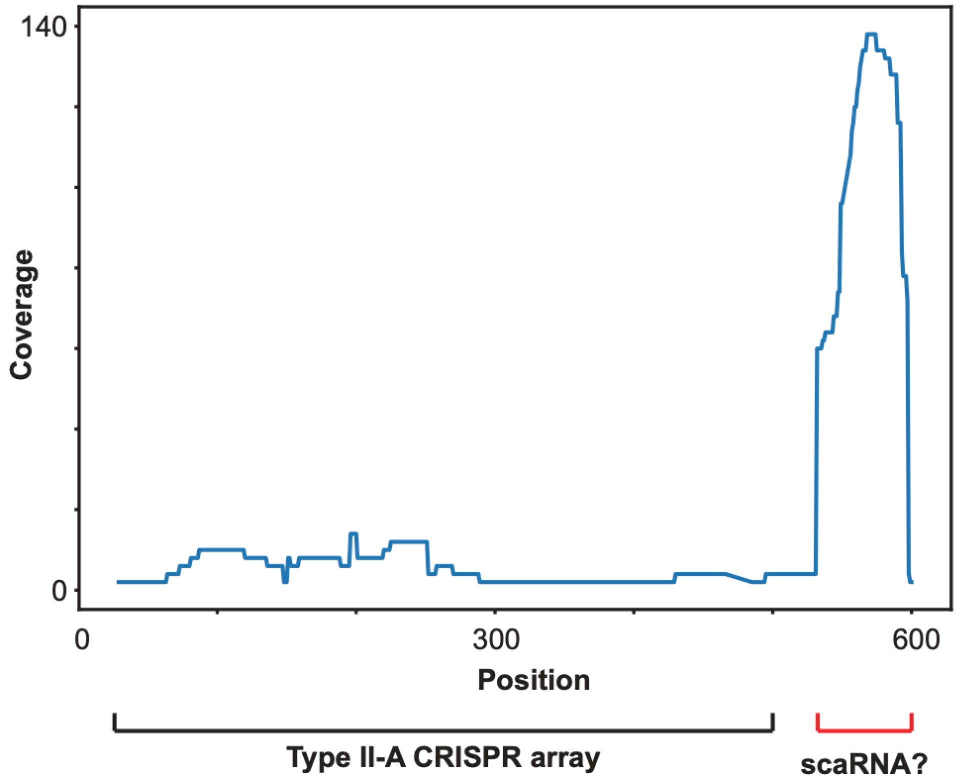

Fig S1. Small RNA coverage graph for the type II-A CRISPR array.

Coverage of small RNA sequencing reads mapped to the CRISPR array and potential small CRISPR-associated RNA (scaRNA) region of *L. salivarius* NCK 1355.

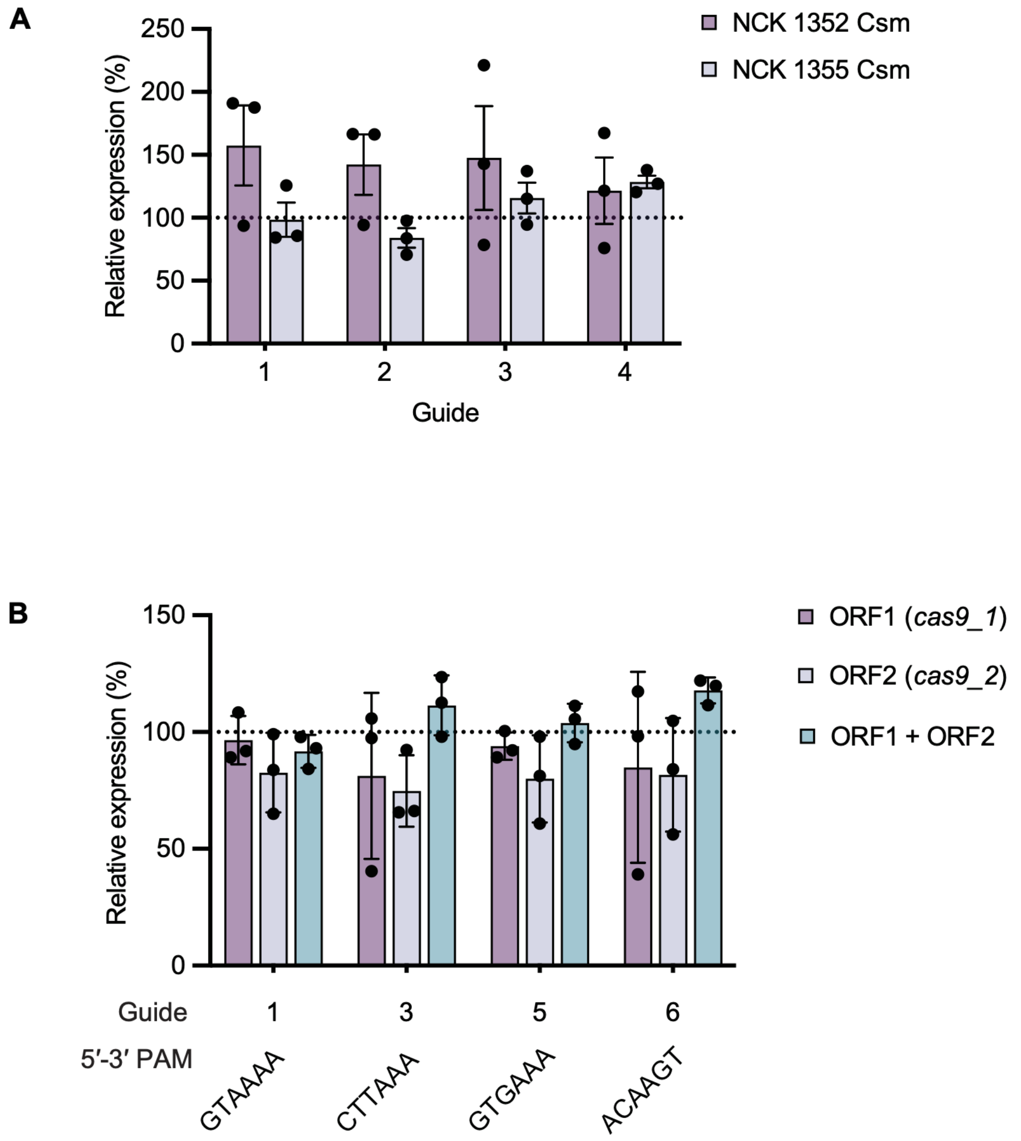

Fig S2. Cell-free transcription-translation assay results for Csm complex pre-expression and split NCK 1355 *cas9* coding sequences.

(A) Relative GFP expression is shown for experiments in which the type III-A CRISPR and Csm complex components were pre-expressed before pTXTL-P70a-deGFP addition. (B) Relative GFP expression is shown for experiments in which the separate NCK 1355 *cas9* coding sequences were tested individually or together, where each coding sequence was expressed from a separate plasmid.

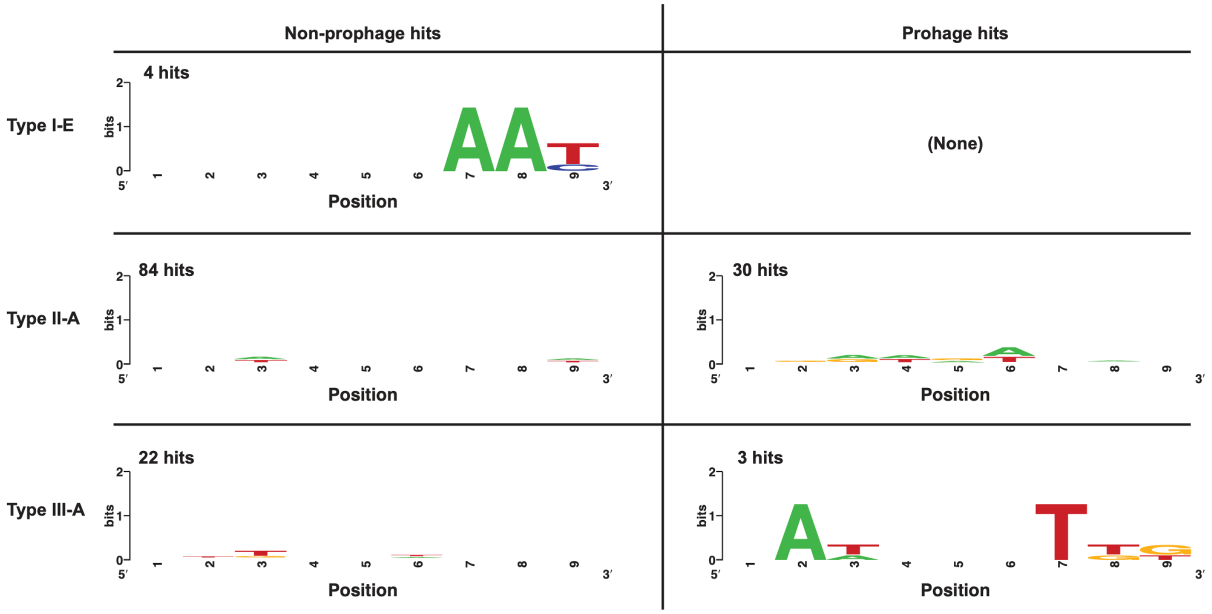

Fig S3. Flanking sequence alignments for self-targeting spacer target sites in predicted prophage and non-prophage elements.

A WebLogo is provided for the putative PAM (type I-E, type II-A) or anti-tag (type III-A) regions for self-targeting spacer target sites. The data is divided between hits within predicted non-prophage or prophage regions. The number of sequences ("hits") used for each WebLogo is provided.
